# Microstructural Control of Soluble Acene Crystals for Field-Effect Transistor Gas Sensors

**DOI:** 10.3390/nano12152564

**Published:** 2022-07-26

**Authors:** Jung Hun Lee, Jeong Hwan Chun, Hyun-Jong Chung, Wi Hyoung Lee

**Affiliations:** 1 Department of Materials Science and Engineering, Seoul National University, Seoul 08826, Korea; charmingh2@snu.ac.kr; 2 Department of Organic and Nano System Engineering, Konkuk University, Seoul 05029, Korea; jswjdghks@naver.com; 3 Department of Physics, Konkuk University, Seoul 05029, Korea; hjchung@konkuk.ac.kr; 4 Division of Chemical Engineering, Konkuk University, Seoul 05029, Korea

**Keywords:** gas sensors, organic field-effect transistors, soluble acene crystals, microstructure, sensitivity, selectivity

## Abstract

Microstructural control during the solution processing of small-molecule semiconductors (namely, soluble acene) is important for enhancing the performance of field-effect transistors (FET) and sensors. This focused review introduces strategies to enhance the gas-sensing properties (sensitivity, recovery, selectivity, and stability) of soluble acene FET sensors by considering their sensing mechanism. Defects, such as grain boundaries and crystal edges, provide diffusion pathways for target gas molecules to reach the semiconductor-dielectric interface, thereby enhancing sensitivity and recovery. Representative studies on grain boundary engineering, patterning, and pore generation in the formation of soluble acene crystals are reviewed. The phase separation and microstructure of soluble acene/polymer blends for enhancing gas-sensing performance are also reviewed. Finally, flexible gas sensors using soluble acenes and soluble acene/polymer blends are introduced, and future research perspectives in this field are suggested.

## 1. Introduction

Solution-processed small-molecule semiconductors (so-called soluble acenes) have attracted much attention as alternative field-effect transistors (FETs) for flexible switching elements in displays and sensors [1,2,3,4,5,6,7,8]. Control of the crystalline microstructure during the solution processing of soluble acene is critical for its desired function in FETs and sensors [9,10,11,12,13,14,15]. Typically, highly crystalline films are desirable for the effective π–π stacking of conjugated acene moieties and the corresponding lateral transport of charge carriers in FETs. Although the fabrication of uniform and large-area single-crystal films is a target goal for achieving the best performance in FETs, solution processing of soluble acene typically leads to a polycrystalline film with defects inside the film. Accordingly, the fine control of solvent evaporation during the formation of crystals is necessary. Several well-written review articles focusing on the microstructural and morphological control during solvent evaporation in the casted soluble acene solution are available [9,10,11,16,17,18,19,20,21]. Strategies, such as the control of solution composition, printing parameters (for example, spin speed in spin-casting and jetting condition in inkjet printing), and surface energy of the substrate, have been well documented in recent publications for high-performance soluble acene FETs [9,16,17,18,22,23,24].

In addition to the performance of FETs (for example, field-effect mobility and on–off current ratio threshold voltage), sensors require other performance parameters [25,26,27,28,29,30,31]. FET-based gas sensors particularly require a diffusion pathway for the active semiconductor channel for gas molecules to reach the semiconductor-dielectric interface. Note that the semiconducting layer near the semiconductor-dielectric interface is the region where most of the field-effect charge carriers pass through in a given gate bias of FETs. The gas molecules affect these charge carriers at the interface, leading to a current change. The current change is monitored at the constant source-drain bias condition in FETs, and sensitivity, recovery, selectivity, and stability are the required performance parameters in FET-based gas sensors [26,27,32,33,34].

In FET-based gas sensors, the formation of defects (such as grain boundaries, crystal edges, and pores) is key to guaranteeing a diffusion pathway for sensitive gas detection [15,23,26,35,36,37]. However, review articles focusing on the defect engineering of soluble acene crystals with a particular focus on the employed strategies are currently unavailable, although there have been many reports on controlling these defects in soluble acene FET-based gas sensors. The use of soluble acene in the semiconducting layers of FET-based gas sensors has several advantages. First, soluble acene FETs can be used for detecting environmentally harmful gases (such as nitrogen dioxide and ammonia) and healthcare-related exhaled gases (such as formaldehyde and acetone) with a detection limit as low as ppb. Second, selectivity to the target gas is feasible when using a specific soluble acene, which can be obtained by the synthesis of a new soluble acene. Here, the manipulation of specific interactions between the soluble acene and target gas molecules is required. Third, the printing technique can be utilized to fabricate large-area/flexible gas sensors on plastic substrates as it is better than a vacuum-based evaporation tool for reducing costs and increasing manufacturing speed [23,38,39,40,41,42]. Typically, a polymer binder is added to a soluble acene solution to fabricate printing-based, flexible gas sensors. The polymer can increase the solution viscosity while reducing the dewetting of soluble acene crystals. Here, inducing vertical phase separation in soluble acene/polymer blends is also necessary for fabricating FETs. Readers can read recent review papers focusing on obtaining high field-effect mobility in FETs based on soluble acene/polymer blends [17,18,43,44,45,46,47,48,49]. Instead of introducing strategies for vertical phase separation, this review focuses on introducing key concepts in the microstructural control of soluble acene/polymer blends for high-performance gas sensors.

This focused review paper introduces several representative works on the microstructural control in soluble acene and soluble acene/polymer blends for high-performance FET-based gas sensors. Section 2 introduces the working principle of the soluble acene FET-based gas sensor. Section 3 reviews the control of the soluble acene microstructure to enhance the gas-sensing performance. Section 4 reviews the microstructural control of soluble acene/polymer blends for improving the gas-sensing performance. Section 5 introduces representative studies on flexible soluble acene gas sensors. Finally, the conclusions and future research perspectives are presented.

## 2. Soluble Acene FET-based Gas Sensors: Working Principle

**Figure 1:**
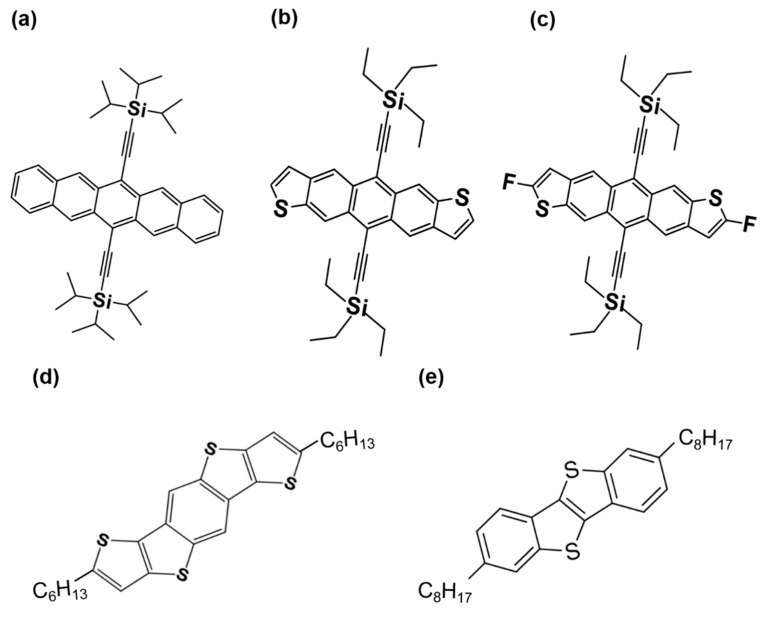
Chemical structures of prototypical soluble acenes: (**a**) 6,13-bis(triisopropylsilylethynyl)pentacene (TIPS-pentacene), (**b**) 5,11-bis(triethylsilylethynyl)anthradithiophene (TES-ADT), (**c**) 2,8-difluoro-5,11-bis(triethylsilylethynyl)anthradithiophene (diF-TES-ADT), (**d**) 2,7-dihexyl-dithieno[2,3-d;2′,3′-d′]benzo[1,2-b;4,5-b′]dithiophene, and (**e**) 2,7-dioctylbenzothieno[2,3-b]benzothiophene (C8-BTBT).

This review uses the word “soluble acene” to mention solution-processed small molecular semiconductors. Typically, the semiconducting properties of organic molecules are induced by extending conjugation in fused acenes or hetero-acenes [50,51]. However, fused acene or hetero-acene (e.g., pentacene) are not soluble in common solvents, although some studies solubilized pentacene with harsh chemical treatments [52,53,54]. The addition of solubilizing groups to the six and 13 positions in pentacene can increase its solubility while changing the packing motif from herringbone stacking to co-facial brick wall stacking (Figure 1a) [55]. Figure 1 shows the chemical structures of representative soluble acenes, (e.g., 6,13-bis(triisopropylsilylethynyl)pentacene (TIPS-pentacene)). In addition to the commonly used spin-coating or inkjet printing, solution-shearing-assisted bar coating leads to a high-performance TIPS-pentacene FET with field-effect mobility exceeding 4.6 cm^2^/Vs [56,57,58]. The addition of triethylsilylethynyl groups to the anthradithiophene backbone is also an effective method for increasing both solubility and solid-state ordering (Figure 1b) [59]. Interestingly, a 5,11-bis(triethylsilylethynyl)anthradithiophene (TES-ADT) film was crystallized by solvent vapor annealing, and millimeter-sized TES-ADT spherulites were easily fabricated using this method [60,61,62]. The further addition of fluorine atoms to TES-ADT led to exceptionally small π–π stacking due to fluorine-fluorine interactions (Figure 1c) [63]. Accordingly, 2,8-difluoro-5,11-bis(triethylsilylethynyl)anthradithiophene (diF-TES-ADT) exhibited an ultrafast crystallization capability during solution processing. High mobility exceeding 5 cm^2^/Vs has been reported in FETs based on diF-TES-ADT/polymer blends [6,64,65,66]. Instead of bulky silylethynyl groups, alkyl chains (such as hexyl and octyl) can also be attached to the edges of the acene backbone to enable solution processability (Figure 1d,e). 2,7-Dihexyl-dithieno[2,3-d;2′,3′-d′]benzo[1,2-b;4,5-b′]dithiophene (C6-DTBDT) and 2,7-dioctylbenzothieno[2,3-b]benzothiophene (C8-BTBT) are examples that show excellent field-effect mobilities in the application of FETs [49,67,68,69]. 

**Figure 2:**
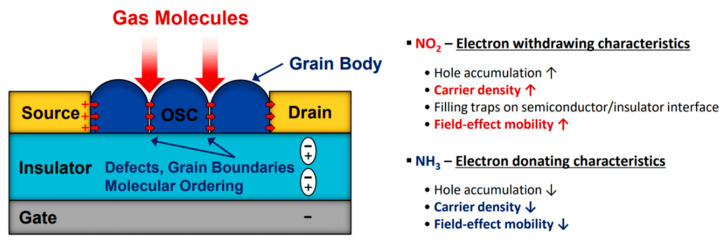
Gas sensing mechanism of organic-effect transistor gas sensors. OSC represents organic semiconductor.

Figure 2 illustrates the gas-sensing mechanism when these soluble acenes are used as the active layer in FET gas sensors. When a gate bias is applied in FETs, the polarization of the insulator leads to an accumulation of charge carriers at the semiconducting layer near the semiconductor-insulator interface. Because all the soluble acenes in Figure 1 are operated in the p-type mode, a negative gate bias is applied, and hole carriers are generally accumulated at the semiconducting layer. When gas molecules penetrate the semiconducting layer near the semiconductor-insulator interface, hole carriers increase (or decrease) depending on the type of gas molecules [25,70,71,72]. In the case of oxidizing gases, such as NO_2_, the electron-withdrawing character of the gas induces hole accumulation (that is, an increase in carrier density). Thus, the source-drain current increases upon exposure to the oxidizing gas. In contrast, the electron-donating character of reducing gases, such as NH_3_, induces hole depletion (that is, a decrease in carrier density), leading to a decrease in the source-drain current. The change in the field-effect mobility can also be monitored in FET gas sensors. If the semiconductor is not a perfect single crystal (this is the typical case in soluble acene films manufactured using solution processing), the trap filling behavior in the oxidizing gas leads to an increase in the field-effect mobility. In contrast, it decreases upon exposure to a reducing gas. However, the change in mobility is also affected by the scattering of charge carriers by gas molecules. In that case, the field-effect mobility decreases in the presence of reducing/oxidizing gases. The charge–dipole interaction between the semiconductor and gas molecules could also change the mobility of gas sensors. Accordingly, changes in the electrical performance of FETs depend on the specific soluble acene type and target gas molecules.

## 3. Control of Soluble Acene Microstructure for Gas Sensors

Soluble acene microstructure could be manipulated by the careful control of solvent evaporation during solution processing (e.g., drop-casting, spin-coating, inkjet printing, bar coating). Especially, the use of solvent with a high boiling point is preferable for inducing slow evaporation of solvent, thereby increasing crystallinity with structural perfectness. However, soluble acene with low film-forming capability triggers thin-film dewetting during solution processing. Thus, solvent with appropriate vapor pressure and viscosity is used in solution processing such as spin-coating and bar coating. Because crystalline microstructure (e.g., crystallinity, grain boundary, misorientation) of soluble acene is highly dependent on the processing condition, fine control solvent evaporation is indispensable for the proper functions in FETs and sensors. Because several well-written review articles are available in the morphological and structural control of soluble acene [9,10,11,16,17,18,19,20,21], control of the microstructure focusing on gas sensing performance is introduced in this section. In addition, the gas sensing mechanism in the representative works is reviewed by considering the specific interaction between soluble acene and gas molecules.

**Figure 3:**
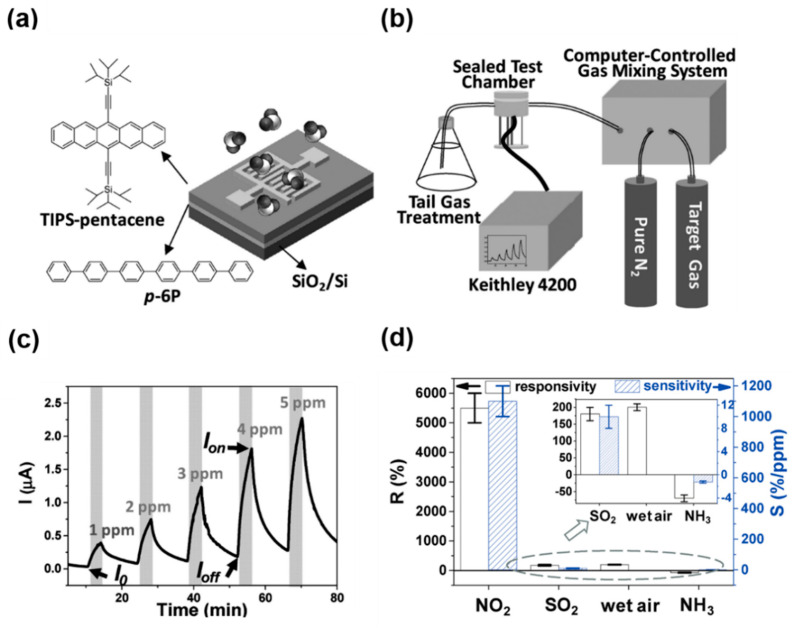
(**a**) Device configuration of TIPS-pentacene FET sensors, (**b**) sensor setup system, (**c**) dynamic response of the TIPS-pentacene/para-sexiphenyl (*p*-6P) film upon periodic exposure to NO_2_ (1–5 ppm), and (**d**) responsivity (R) and sensitivity (S) to different gases (NO_2_, SO_2_, wet air, and NH_3_) [74]. Copyright 2017 Wiley.

Soluble acene is composed of an aromatic conjugated core (e.g., fused acene, hetero-acene) and a solubilizing group (e.g., aliphatic alkyl chain) (Figure 1). In soluble acene FET gas sensors, it is important to examine the specific interaction between soluble acene and gas molecules from the prototypical soluble acene, TIPS-pentacene. Because TIPS-pentacene is oriented with silylethynyl groups on the substrate surface (Figure 1a) [55], the adsorption of gas molecules on TIPS-pentacene may be weaker than pentacene without insulating silylethynyl groups. The pentacene backbone cannot interact directly with the gas molecules in TIPS-pentacene. However, a recent report by Wang et al., revealed that TIPS-pentacene FETs could detect ppm levels of NO_2_ better than pentacene FETs [73]. Figure 3a shows the device structure of TIPS-pentacene FET sensors. Para-sexiphenyl (*p*-6P) was used as the buffer layer to obtain a terrace-like TIPS-pentacene film. Although TIPS-pentacene was thermally evaporated on the *p*-6P film, the sensing modality of TIPS-pentacene could be measured using the gas testing setup (Figure 3b). Dynamic tests with periodic exposure to NO_2_ showed that the TIPS-pentacene sensor exhibited excellent sensitivity (>1000%/ppm), recovery (>90%), and reproducibility (Figure 3c). When cross-sensitivity was measured with different gases (Figure 3d), the TIPS-pentacene sensor exhibited selective detection of NO_2_. The responsivities and sensitivities to SO_2_, wet air, and NH_3_ were low. Interestingly, the TIPS-pentacene FETs exhibited better sensing performance than the pentacene FETs. The combined effect of low intrinsic conductivity and efficient charge transport ability in TIPS-pentacene films leads to a high gas-on/gas-off conductivity, resulting in excellent sensitivity toward NO_2_. A similar performance enhancement of the TIPS-pentacene sensor toward NH_3_ compared to pentacene sensor has also been reported [74].

**Figure 4:**
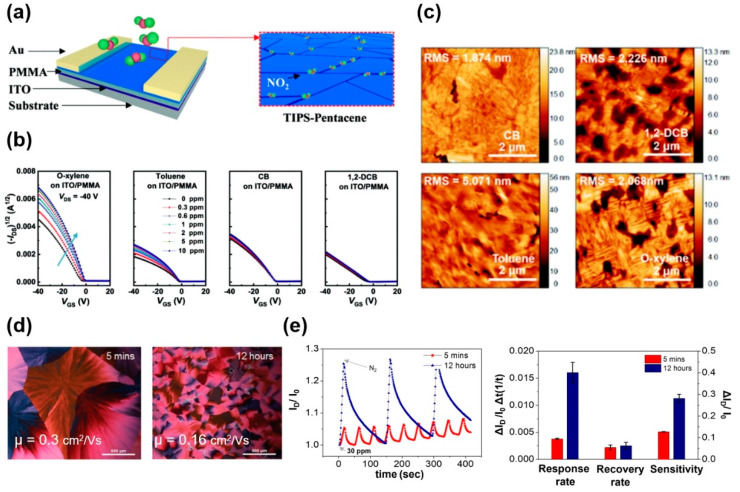
(**a**) The device structure and sensing mechanism of TIPS-pentacene sensors showing diffusion of NO_2_ through the grain boundaries, (**b**) the change in the transfer curves for the o-xylene, toluene, chlorobenzene (CB), and 1,2-dichlorobenzene (1,2-DCB)-processed TIPS-pentacene FETs upon NO_2_ exposure at various concentrations, (**c**) atomic force microscopy images of TIPS-pentacene films processed with different solvents [75]. Copyright 2019 Royal Society of Chemistry. (**d**) Polarized optical microscopy images of the solvent vapor annealed TES-ADT films whose solutions were mixed for 5 min or 12 h, and (**e**) response curves and summarized performance of TES-ADT FETs upon sequential NO_2_ and N_2_ exposure [76]. Copyright 2017 Wiley.

Because the diffusion of gas molecules into the semiconducting layer near the semiconductor-dielectric interface is important, defects, grain boundaries, and molecular ordering in soluble acene films affect the gas sensing performance of soluble acene FET-based gas sensors. Thus, it is important to control the soluble acene microstructure during solution processing. Shao et al., examined the effect of crystallinity and grain boundaries on the NO_2_ sensing performance of TIPS-pentacene FETs using different types of processing solvents (Figure 4a) [75]. When four different types of solvents (namely, o-xylene, toluene, chlorobenzene (CB), and 1,2-dichlorobenzene (1,2-DCB)) were used, the spin-cast films exhibited different morphologies and microstructures. Figure 4b shows the NO_2_ sensing performance for the different processing solvents. Upon exposure to NO_2_, the hole-carrier density and field-effect mobility increased. The responses were ordered as follows: o-xylene > toluene > CB > 1,2-DCB. Atomic force microscopy images and X-ray diffraction results indicated that TIPS-pentacene from o-xylene exhibited a highly crystalline structure with a high density of grain boundaries (Figure 4c). In contrast, the CB sample exhibited a lower grain boundary density. Thus, TIPS-pentacene FETs fabricated from CB exhibited low sensitivity, regardless of their high field-effect mobility. The 1,2-DCB sample had a loosely connected microstructure with the lowest crystallinity, degrading the field-effect mobility and sensitivity. Using the TIPS-pentacene film fabricated from o-xylene, the limit of detection of NO_2_ could be lowered to 1.93 ppb. Seo et al., found that the gas-sensing performance was enhanced by increasing the grain boundary density in TES-ADT spherulites [76]. The grain boundary density was regulated by changing the mixing time of the TES-ADT solution [77,78]. Subsequent solvent vapor annealing of the spin-cast TES-ADT films led to the formation of TES-ADT spherulites with different grain boundary densities (Figure 4d). A mixing time of 5 min resulted in a large grain size and a corresponding high field-effect mobility of 0.3 cm^2^/Vs. However, a mixing time of 12 h led to a smaller grain size and moderate field-effect mobility of 0.16 cm^2^/Vs. The mixing-induced self-aggregation behavior of TES-ADT molecules triggers aggregates in the spin-cast TES-ADT film, and subsequent solvent vapor annealing leads to a high nucleation density and corresponding small spherulites with a high grain boundary density. The response rate and sensitivity of the TES-ADT film with a high grain boundary density (sample of 12 h) exhibited significantly better performance, notwithstanding the low field-effect mobility (Figure 4e). Similarly, the increase in grain boundaries by vertical annealing or solvent vapor annealing could also be an effective method for increasing the sensitivity to NO_2_ [79,80]. From these results, grain boundaries provide a pathway for the target gas molecules to reach the semiconductor-insulator interface.

**Figure 5:**
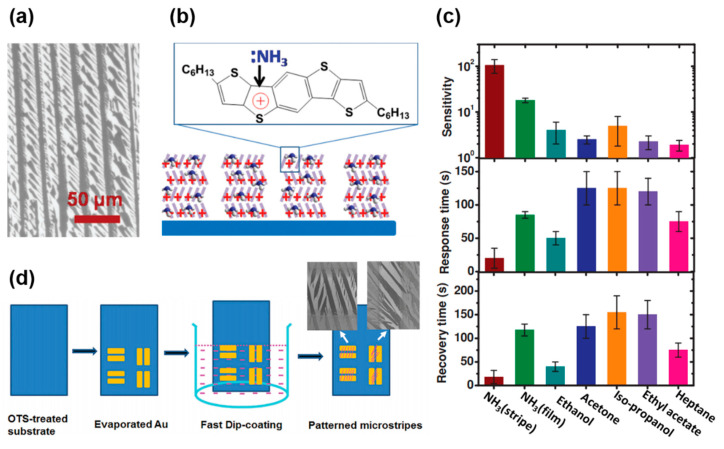
(**a**) Scanning electron microscopy image of C6-BTBDT microstripes, (**b**) sensing mechanism of C6-BTBDT with NH_3_, (**c**) sensitivity, response time, and recovery time of C6-BTBDT FET sensors according to the target gas molecules [81]. Sensitivity is defined as *I*_NH3-off_/*I*_NH3-on_. Copyright 2013 Wiley. (**d**) Fabrication procedure and scanning electron microscopy images of C6-BTBDT microstripes on Au source/drain electrodes [82]. Copyright 2016 Royal Society of Chemistry.

The diffusion pathway for the gas molecules can also be enlarged by fabricating soluble acene stripes. Gas molecules easily reach the semiconductor-dielectric interface through these crystal edges. Li et al., demonstrated that ultrathin C6-DTBDT microstripes could be a good sensing platform for adsorption, diffusion, interaction, and desorption activities [81]. They fabricated C6-DTBDT microstripes using dip coating (Figure 5a), and the sensor based on C6-DTBDT microstrips exhibited an excellent response toward NH_3_. The electrostatic interaction between the electron-deficient thiophene unit and electron-rich NH_3_ led to the adsorption of NH_3_, thereby inducing traps and de-doping inside the channel (Figure 5b). The combined effect of de-doping and dipole-charge interactions led to an abrupt decrease in the channel current. Here, the form and microstripes were better than the film because the interaction could be facilitated by the efficient pathway between the microstripes (Figure 5c). Although the sensor responded to other analytes, it exhibited the best performance (for example, high sensitivity and low response/recovery times) for NH_3_. C6-DTBDT microstripes across Au source/drain electrodes were fabricated using the wetting-dewetting concept with evaporated Au electrodes and octadecyltrichlorosilane-treated SiO_2_ (Figure 5d) [82]. It was possible to fabricate C6-DTBDT microstripes across the channel region, and the sensor exhibited an excellent response to NH_3_.

**Figure 6:**
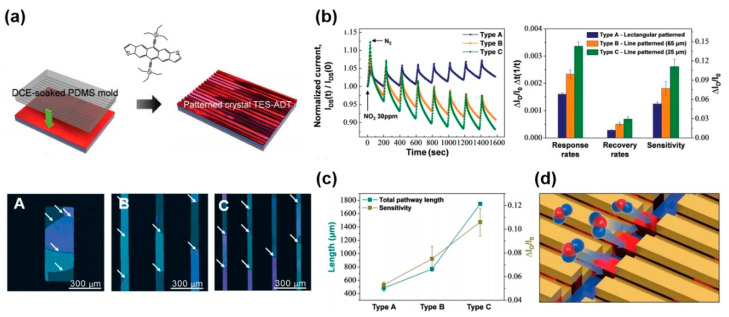
(**a**) Fabrication procedure of TES-ADT microstripes using a polydimethylsiloxane (PDMS) mold and the types of fabricated microstripes: (**A**) rectangular patterns of 200 µm width, (**B**) line pattern of 65 µm width, and (**C**) line patterns of 25 µm width. The white arrows indicate the grain boundary. (**b**) gas-sensing characteristics and summarized performance according to the types of microstripes, (**c**) comparison curve of the sensitivity versus pathway length (sum of grain boundary length and edge length), and (**d**) schematic showing gas diffusion in TES-ADT microstripes [83]. Copyright 2020 Wiley.

The patterning (such as rectangle or line) of soluble acene is important for guaranteeing the diffusion pathway for target gas molecules. Kwak et al., systematically examined the relationship between the dimensions of rectangular/line patterns and the gas-sensing performance [83]. They used a rectangular/line-patterned polydimethylsiloxane (PDMS) mold to fabricate TES-ADT crystal arrays (Figure 6a, top). The contact region in the TES-ADT film could be etched by the solvent-soaked PDMS mold, whereas solvent vapor-assisted crystallization was facilitated in the non-contact region [15,84,85]. Changing the pattern dimensions in the PDMS mold, it was possible to fabricate TES-ADT crystal arrays with different widths (Figure 6a, bottom). The gas-sensing performance was heavily dependent on the pattern type. With the narrowest line width, Type C exhibited the highest performance (such as response rate, recovery rate, and sensitivity) for NO_2_ (Figure 6b). Because the total pathway length for gas diffusion can be estimated from the sum of the grain boundary and edge lengths, the sensitivity is proportional to the total pathway for gas diffusion (Figure 6c). From these results, it was concluded that the ultrathin microstripe pattern provides an efficient gas-sensing platform for reversible gas adsorption and desorption (Figure 6d).

## 4. Microstructural Control of Soluble Acene/Polymer Blends for Gas Sensors

**Figure 7:**
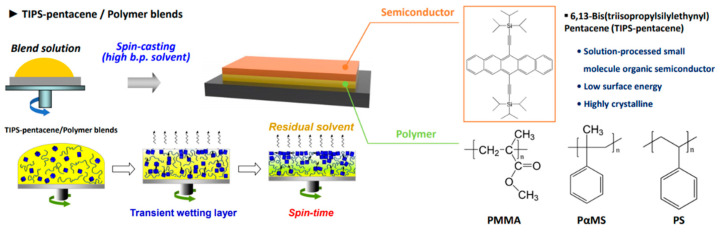
Schematic showing phase-separation behaviors of TIPS-pentacene/polymer blends during the evaporation of the residual solvent [86]. Copyright 2012 Wiley. Typically, a vertically phase-separated structure consisting of TIPS-pentacene at the top and polymer at the bottom is formed on SiO_2_/Si.

Because soluble acene has a low film-forming capability, polymer binders are typically added to prepare soluble acene solutions, and spin-casting, dip-coating, inkjet printing, and bar coating are applied to fabricate soluble acene/polymer blend films [17,39,43,44,45]. In addition to the insulating binder polymers, poly(triaryl amine) (PTAA) with the highest occupied molecular orbital level similar to that of soluble acene is used as the counterpart polymer in the preparation of the blend solution [6,64]. However, there have not been any reports on soluble acene/PTAA blends in gas sensor applications; therefore, papers on soluble acene/PTAA blends are excluded in this review which focuses on FET gas sensors. As the charge carrier path is substantially lateral to the substrate surface, the formation of a vertically phase-separated structure is indispensable. The authors reviewed well-written review articles that introduce recent papers dealing with phase separation in soluble acene/insulating polymer blends [17]. For gas sensor applications, the active layer should be exposed to ambient air; therefore, the bottom-gate structure is preferable to the top-gate structure. Figure 7 shows a schematic of the phase-separation behavior of TIPS-pentacene/polymer blends during the evaporation of the residual solvent. Insulating polymers (such as poly(methyl methacrylate) (PMMA), poly(alpha-methylstyrene) (PαMS), and polystyrene (PS)) can be used as binder polymers, which increase the solution viscosity and reduce the dewetting of soluble acene. The TIPS-pentacene-top/polymer-bottom structure is spontaneously induced during spin-casting, mainly because of the lower surface energy of TIPS-pentacene compared to its counterpart polymer. Here, the residual solvent at a given spin time determines the phase-separated structure and crystallization behavior of the soluble acene (Figure 7, bottom schematic) [13,86].

**Figure 8:**
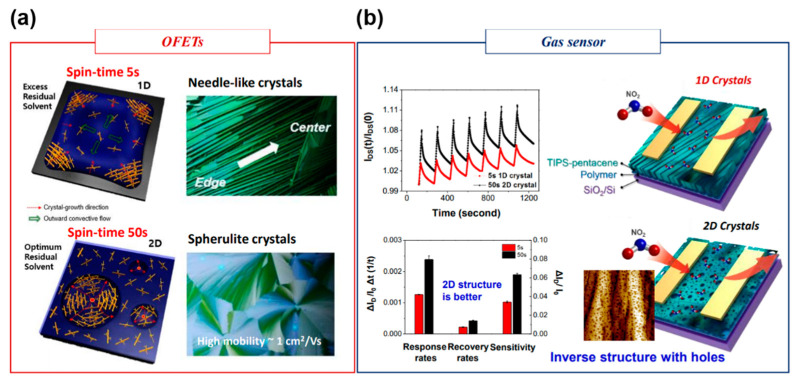
Spin-coating time (5 s or 50 s) governs the 1D versus 2D growth of TIPS-pentacene on a polymer, affecting the performance of FETs and gas sensors [37]. Copyright 2018 Wiley. (**a**) Mechanism and polarized optical microscopy images of TIPS-pentacene crystals and (**b**) NO_2_ sensing performance and schematics showing the gas-sensing mechanism [37]. Copyright 2019 Nature Publishing Group.

Lee et al., examined the phase separation and structural development of soluble acene/polymer blends by changing the spin time of a spin-casting blend solution [13]. 1,2-dichlorobenzene with a high boiling point (that is, low solvent evaporation rate) was used to amplify the effect of the residual solvent (Figure 7). A vertically phase-separated structure consisting of a soluble acene-top and polymer-bottom was induced, regardless of the spin time. In a short spin time of 5 s, the excess residual solvent resulted in the flow-induced growth of needle-like one-dimensional (1D) crystals from the edge to the center position (Figure 8a). In contrast, the optimum residual solvent at a spin time of 50 s resulted in two-dimensional (2D) spherulite crystals. The 2D crystals at 50 s exhibit significantly higher mobility (approximately 1 cm^2^/Vs) in FET applications than the 1D crystals at 5 s because of the higher crystal perfection and coverage of soluble acene crystals on the phase-separated insulating polymer. The response rate, recovery rate, and sensitivity toward NO_2_ of the 2D structure were better than those of the 1D structure (Figure 8b) [37]. Although the 1D microstructure provides a microscale route for gas adsorption/desorption, the inverse structure in the 2D crystals has many nanoscale holes (Figure 8b, bottom image). A porous structure with a lower film thickness and nanoscale holes in 2D crystals is preferable for gas diffusion, whereas higher field-effect mobility facilitates fast detection in FET gas sensors. It should be emphasized that the 2D structure was better than the 1D structure in the proposed experimental system. Although other results opposite to this trend are possible, a porous structure with many holes provides an excellent sensing platform for FET-based gas sensors.

**Figure 9:**
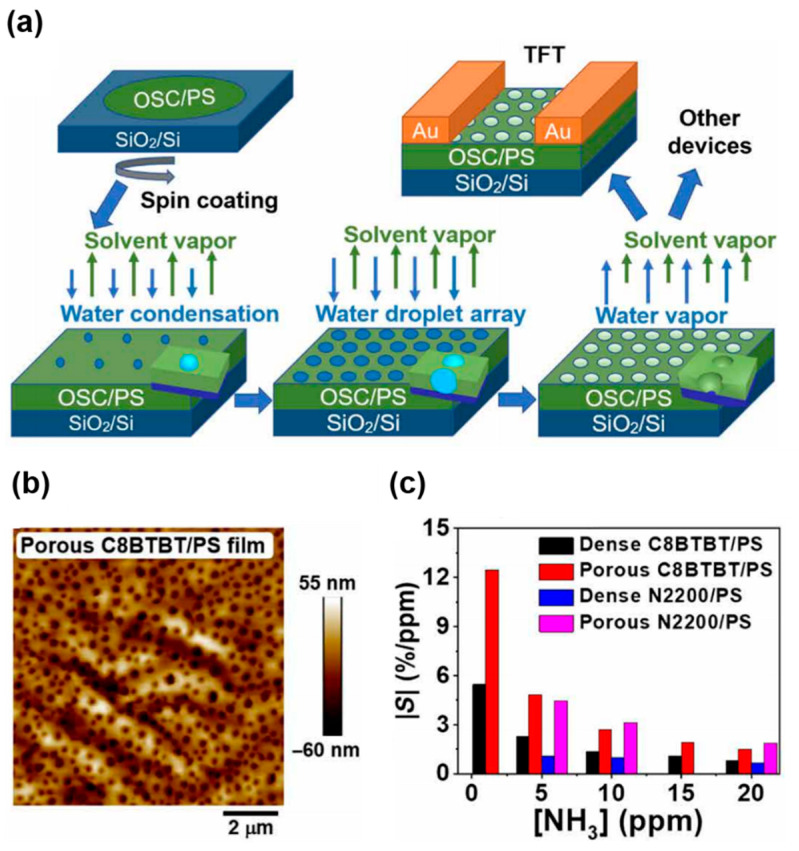
(**a**) Fabrication procedure for a porous organic semiconductor film, (**b**) atomic force microscopy image of a porous C8-BTBT/polystyrene (PS) film, and (**c**) sensitivities of FETs-based gas sensors based on dense/porous C8-BTBT/PS and poly[*N,N′*-bis(2-octyldodecyl)-naphthalene-1,4,5,8-bis(dicarboximide)-2,6-diyl]-alt-5,5′-(2,2′-bithio-phene) (N2200)/PS films toward different NH_3_ concentrations (ppm) [87]. Sensitivity is defined as *S* = [(*I*_Gas_ − *I*_0_)/*I*_0_]/[*NH*_3_] × 100%. Copyright 2020 AAAS.

Although porous structures have been proposed for FET-based gas sensors, a general route for fabricating porous structures (including soluble acene films) is necessary. Zhang et al., proposed a methodology for fabricating porous semiconducting films with organic semiconductor/PS blends (Figure 9a) [87]. In the so-called breath figure method, water condensation under high humidity conditions (approximately 60%) and subsequent evaporation of water under thermal annealing led to porous films. Figure 9b shows a C8-BTBT/PS film fabricated by the breath figure method. Porous structures were easily fabricated with other blend solutions (such as p-type poly(3-hexylthiophene)/PS, n-type poly[*N,N′*-bis(2-octyldodecyl)-naphthalene-1,4,5,8-bis(dicarboximide)-2,6-diyl]-alt-5,5′-(2,2′-bithio-phene) (N2200)/PS). Figure 9c compares the gas-sensing properties of dense and porous blend films. Porous films exhibit considerably better sensitivities to NH_3_ than dense films. This result indicates that a more accessible morphology with nanoscale holes in the active layer is advantageous for guaranteeing a diffusion pathway in FET gas sensors.

**Figure 10:**
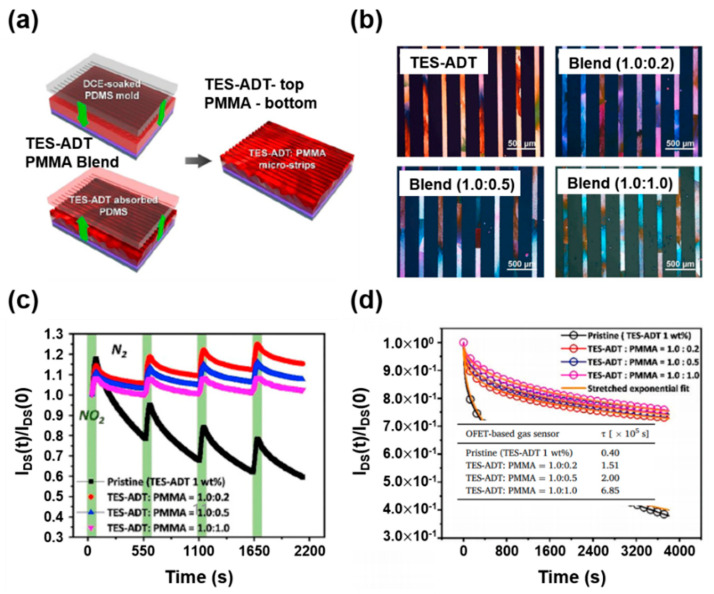
(**a**) Fabrication procedure of TES-ADT/poly(methyl methacrylate) (PMMA) microstripes using a PDMS mold, (**b**) polarized optical microscopy images of the fabricated microstripes from TES-ADT and TES-ADT/PMMA blends, (**c**) gas-sensing characteristics toward NO_2_ according to the types of microstripes, and (**d**) gate-bias stabilities at continuous bias conditions (V_GS_ = −20 V, V_DS_ = −10 V) [15]. Copyright 2020 Elsevier.

Soluble acene/polymer blends are beneficial in terms of long-term device stability (such as environmental and gate-bias stability). In particular, the gate-bias instability of soluble acene FETs can be reduced by incorporating an insulating polymer, which is easily achieved by blending with an insulating polymer [88]. The FET-based gas sensors are also operated under continuous bias conditions. Accordingly, the gate-bias screens the hole carriers in p-type organic semiconductors, leading to on-current decay and a negative shift in the threshold voltage. It is necessary to decrease the gate-bias instability in FET gas sensors using several strategies. Kwak et al., enhanced the device stability of TES-ADT microstripe sensors using TES-ADT/PMMA blends [15]. A patterning method similar to Figure 6a was used, and spin-casting of the TES-ADT/PMMA blend solution and subsequent stamping with a solvent-soaked/line-patterned PDMS mold led to highly crystalline TES-ADT-top/PMMA-bottom microcrystal arrays (Figure 10a). Figure 10b shows polarized optical microscopy images of the pristine TES-ADT and TES-ADT/PMMA microstripes with different blend ratios. Although the pristine TES-ADT microstripes exhibit a clearly patterned image, the blend samples show residue in the etched region. Additionally, the proportion of lateral phase separation increased with an increasing PMMA content. Consequently, the patterned TES-ADT/PMMA blend FETs exhibited lower field-effect mobility and sensitivity than the patterned TES-ADT FETs (Figure 10c). However, the patterned TES-ADT/PMMA blend FETs showed a significantly lower on-current decay under the sensor operating conditions. When the gate-bias instabilities were measured, the blend FETs exhibited lower on-current decays and corresponding higher extracted characteristic times (τ) (Figure 10d), indicating that the charge carriers in the mobile states increased with the ratio of PMMA in the TES-ADT/PMMA blends. Incorporating PMMA by blending with PMMA provides a route for decreasing the gate-bias instability in soluble acene FET gas sensors.

## 5. Flexible Soluble Acene Gas Sensors

**Figure 11:**
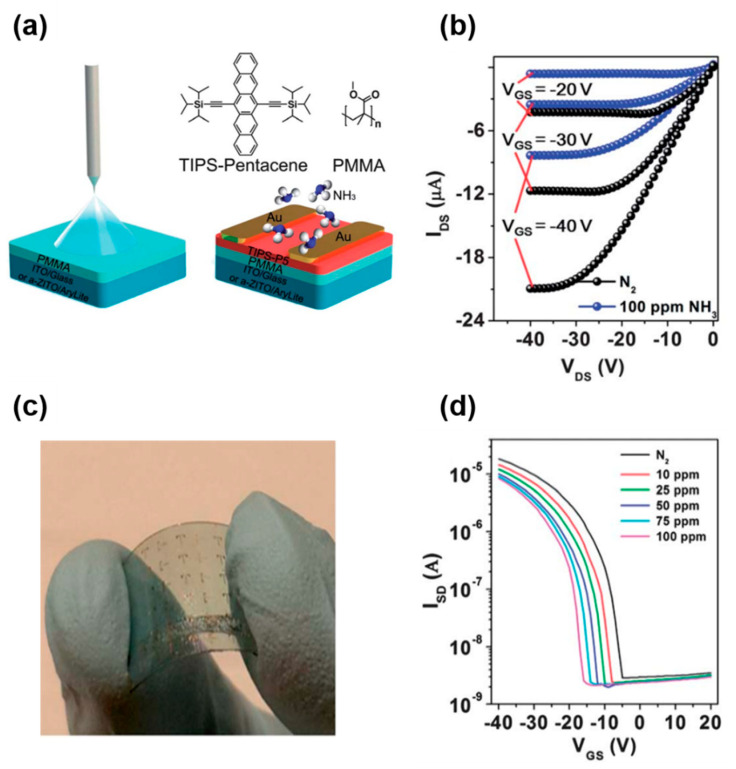
(**a**) Schematic showing the spray coating of TIPS-pentacene on a PMMA/substrate, chemical structures of the used materials, and gas sensor structure of the FETs, (**b**) changes in the output curves before (N_2_) and after target gas (NH_3_, 100 ppm) exposure, (**c**) camera image of a flexible TIPS-pentacene gas sensor, and (**d**) transfer curves of a flexible gas sensor before (N_2_) and after inserting NH_3_ with different concentrations [89]. Copyright 2013 Royal Society of Chemistry.

Soluble acenes are promising candidates for use in flexible printed electronics and sensors. Yu et al., developed a flexible TIPS-pentacene FET gas sensor using spray coating [89]. Spray coating of TIPS-pentacene on a PMMA/substrate led to a highly crystalline TIPS-pentacene film for high-performance OFETs (Figure 11a). Exposure to the NH_3_ target gas resulted in a substantial decrease in the source-drain current in the FETs, mainly due to the electron-donating character of NH_3_ (Figure 11b). Figure 11c shows a flexible gas sensor fabricated on a plastic substrate using spray coating, and the change in the transfer curve under NH_3_ exposure is shown in Figure 11d. As the NH_3_ concentration increased, the turn-on voltage decreased, which is consistent with the decrease in the source-drain current at the given gate and source-drain voltages. Although a flexible gas sensor on a plastic substrate was demonstrated with spray coating, the operating voltage in this study was still high.

**Figure 12:**
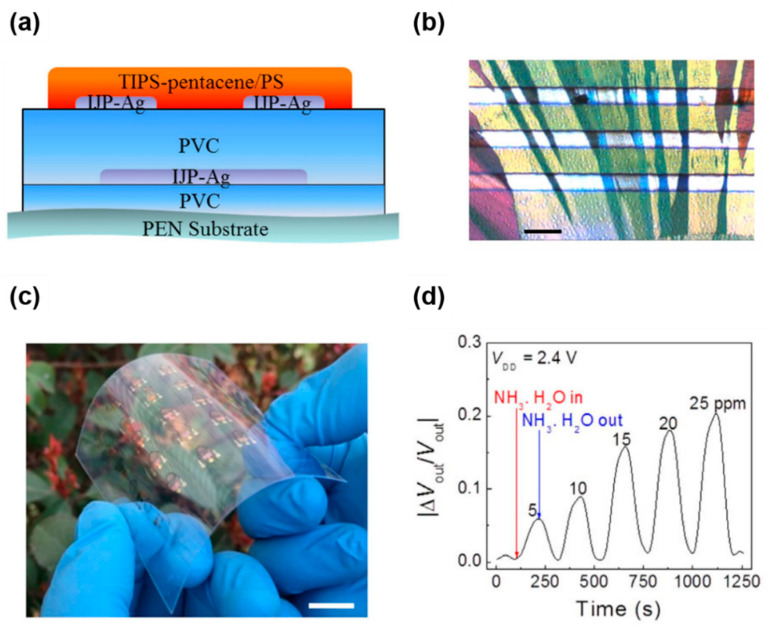
(**a**) Device structure of a flexible FET gas sensor based on a TIPS-pentacene/PS blend, (**b**) optical microscopy image of a TIPS-pentacene/PS blend film, (**c**) camera image of a flexible FET gas sensor, and (**d**) relative change of V_out_ with time upon NH_3_ inlet/outlet with different concentrations [90]. Copyright 2016 Nature Publishing Group.

Feng et al., developed a low-voltage-driven NH_3_ gas sensor based on a TIPS-pentacene/PS blend [90]. Figure 12a displays the device structure of the plastic substrate. They used low-*k* poly(vinyl cinnamate) (PVC) as the gate dielectric and inkjet-printed Ag as the gate and source-drain electrodes. Dropping the TIPS-pentacene/PS blend onto the inclined PVC gate dielectric resulted in the 1D growth of TIPS-pentacene crystals (Figure 12b). Flow-induced 1D growth of TIPS-pentacene on an inclined substrate has previously been reported [91]. In addition to the flexibility shown in Figure 12c, the device exhibited remarkable FET characteristics (such as a low subthreshold slope, low-voltage operation, and operational stability). In particular, the FET exhibited a significant response toward NH_3_ under ambient conditions with a minimum power consumption of 50 nW. Figure 12d shows the readout circuit of the NH_3_ FET gas sensor, which successfully changes the current signal to the voltage output signal (V_out_) using a load resistor. The high signal at the inlet of the ambient NH_3_ and stable background at the outlet of the ambient NH_3_ demonstrated the stable operation of the NH_3_ sensor under ambient conditions.

## 6. Conclusions and Future Perspective

This paper reviews soluble acene-based FET gas sensors, focusing on the microstructural control of soluble acene films and soluble acene/polymer blend films. Soluble acene-based FET sensors exhibit different sensing modalities depending on the type of soluble acene; however, in most cases, they can detect NO_2_ and NH_3_ with dipolar characteristics. Considering the diffusion-limited adsorption/desorption of target gas molecules, the sensitivity can be enhanced by grain boundary engineering, patterning, and generation of pores in the active layer of FET gas sensors. Soluble acene/insulating polymer blends have also been suggested as sensing layers for FETs. The microstructure and vertical phase separation must be finely tuned to enhance the sensitivity, recovery, and stability of the gas sensors. In particular, the growth characteristics of soluble acene on an insulating polymer determine the FET performance and sensor characteristics. Recent studies have also been conducted on flexible soluble acene gas sensors and circuits.

Notwithstanding the introduced studies, the selectivity of the target gas is not easily attainable for soluble acene FET gas sensors. Because NO_2_ is the strongest oxidizing gas in the harmful gas libraries, the sensitivity toward NO_2_ is generally the highest in soluble acene gas sensors. TIPS-pentacene exhibited the highest selectivity for NO_2_. However, it is not easy to select other gases with low oxidation or reducing strength. It should be emphasized that many types of gases exist under atmospheric conditions, which may interfere with the target gas, interrupting the sensing signal from the target gas. Thus, a selective sensing mechanism that manipulates the soluble acene–gas interaction must be developed. Under atmospheric conditions, water and oxygen molecules always affect sensitivity and long-term stability. Thus, a soluble acene gas sensor that is stable under humid atmospheric conditions should be developed. Although a flexible gas sensor has been demonstrated, a soluble acene-based gas sensor with other form factors (such as being stretchable and rollable) must be developed to enable wearable electronics. For applications in wearable electronics, exhale breath sensors employing soluble acene FETs will be particularly useful for the real-time analysis of human diseases.

## Data Availability

Not applicable.
